# Significance of the suture line in cephalopod taxonomy revealed by 3D morphometrics in the modern nautilids *Nautilus* and *Allonautilus*

**DOI:** 10.1038/s41598-021-96611-1

**Published:** 2021-08-24

**Authors:** Amane Tajika, Naoki Morimoto, Neil H. Landman

**Affiliations:** 1 Division of Paleontology (Invertebrates), American Museum of Natural History, Central Park West 79th Street, New York, NY 10024 USA; 2 University Museum, University of Tokyo, 7-3-1 Hongo, Bunkyo-ku, Tokyo, 113-0033 Japan; 3 Laboratory of Physical Anthropology, Graduate School of Science, Kyoto University, Kitashirakawa Oiwake-cho, Sakyo-ku, Kyoto, 606-8502 Japan

**Keywords:** Palaeontology, Taxonomy

## Abstract

Assessing the taxonomic importance of the suture line in shelled cephalopods is a key to better understanding the diversity of this group in Earth history. Because fossils are subject to taphonomic artifacts, an in-depth knowledge of well-preserved modern organisms is needed as an important reference. Here, we examine the suture line morphology of all known species of the modern cephalopods *Nautilus* and *Allonautilus*. We applied computed tomography and geometric morphometrics to quantify the suture line morphology as well as the conch geometry and septal spacing. Results reveal that the suture line and conch geometry are useful in distinguishing species, while septal spacing is less useful. We also constructed cluster trees to illustrate the similarity among species. The tree based on conch geometry in middle ontogeny is nearly congruent with those previously reconstructed based on molecular data. In addition, different geographical populations of the same species of *Nautilus* separate out in this tree. This suggests that genetically distinct (i.e., geographically isolated) populations of *Nautilus* can also be distinguished using conch geometry. Our results are applicable to closely related fossil cephalopods (nautilids), but may not apply to more distantly related forms (ammonoids).

## Introduction

The Cephalopoda are marine mollusks including both extinct (e.g., orthocerids, bactritoids, ammonoids, and belemnoids) and modern taxa (squids, octopuses, and nautiloids). Since their origin in the Cambrian^1^, they have played an important ecological role in the oceans, occupying most likely multiple trophic levels^2–6^. In the fossil record, some cephalopods such as ammonoids are abundant and widely distributed, thus making them ideal model organisms to understand diversification dynamics. One of the apomorphies of externally shelled cephalopods is the chambered conch consisting of a gas-filled phragmocone, providing buoyancy, and a body chamber, accommodating the soft tissue. The phragmocone chambers are septated by partitions called septa; the intersection of the septum and the outer shell wall is known as the suture line. The morphology of septa—and, therefore, the suture line—varies widely among and within cephalopod taxa at different geological times. For example, it is relatively simple in Paleozoic taxa including plectronocerids, oncocerids, and orthocerids, while it is significantly more complex in various clades of ammonoids in the Mesozoic. The complex sutural (septal) morphology is often attributed to constraints related to biomechanics or physiology^7–10^. Most recently, Lemanis et al.^11,12^ concluded that complex septal morphology was likely not an adaptation to increase the resistance against hydrostatic pressure.

The suture line morphology differs at high taxonomic levels in ammonoids and nautiloids. Thus, documenting the morphology of the suture line is traditionally an important part of carrying out taxonomic studies of fossil cephalopods^13,14^. In some cases (e.g., where only a few characters are available), the morphology of the suture line (as well as septal spacing) is an important character to diagnose species^15–18^. However, despite wide application in cephalopod taxonomy, the taxonomic utility of the suture line is still under debate, although paleontologists have attempted to quantitatively examine the morphology of the suture line in ammonoids for decades^19–26^. One of the issues is that, conventionally, the suture line is translated from 3- to 2-dimensional space by hand. This introduces artifacts depending on the researcher. Secondly, fossil material is always subject to time-averaging and post-mortem transport to some degree, introducing another source of variation. Demonstrating the utility of the suture line in cephalopod taxonomy is, thus, of great importance, considering its potential application to a wide range of phragmocone-bearing cephalopods.

Among cephalopod groups, the nautilids *Nautilus* and *Allonautilus*—often dubbed “living fossils”—are the only modern ectocochleate taxa that possess a phragmocone. The study of modern nautilids reduces time-averaging to a minimum (at least at time scales of more than tens of years). In addition, specimens are widely available in museum collections from many localities. While an increasing number of studies have examined the genetics of these animals, shedding new light on their phylogeny and phylogeography^27–32^, our knowledge of the morphology of their shells has not been substantially updated since the 1990’s. Modern nautilids are now protected by the Convention on International Trade in Endangered Species of Wild Fauna and Flora (CITES). Fortunately, modern analytical methods, such as computed tomography, allow for the examination of museum collections in detail without destruction^33,34^.

In this study, we investigate the morphology of the suture line in modern nautilids in detail by applying high-resolution computed tomography and geometric morphometrics. We aim to answer the following questions: (1) To what degree does the morphology of the suture line differ among species of modern nautilids? (2) How does the variation in the morphology of the suture line compare to that of other conch parameters such as conch geometry and septal spacing? (3) Is the suture line a useful diagnostic character for cephalopods compared to other conch parameters? (4) How do our results based on these conch parameters compare to those from molecular data?

## Methods

We studied two specimens of each of the following eight modern nautilid species: *Allonautilus perforatus* (Conrad, 1847) from Indonesia, *Allonautilus scrobiculatus* (Sowerby, 1849) from Papua New Guinea, *Nautilus belauensis* Saunders, 1981 from Palau, *Nautilus macromphalus* Sowerby, 1849 from New Caledonia, *Nautilus pompilius* Linnaeus, 1758 from Papua New Guinea and the Philippines, *Nautilus pompilius suluensis* Habe and Okutani, 1988 from the Philippines, *Nautilus repertus* Iredale, 1944 from Western Australia, and *Nautilus stenomphalus* Sowerby, 1848 from Lizard Island, Australia. Brief descriptions of each of these species are summarized in the Supplementary Note. Most of the specimens were caught live between 1970 and 2010. The sex was recorded for a few individuals (see Table 1 for details). All specimens were identified as nearly to fully mature because all specimens show a crowding of the last few septa and/or exhibit a black band at the aperture^35,36^. Most specimens are housed in the American Museum of Natural History (with the abbreviation AMNH); the specimens of *N. pompilius* from the Philippines are in the National Museum of Nature and Science, Japan (with the abbreviation NMNS PM).

**Table 1:** Details of the examined specimens.

Species	Geographic region	Specimen number	Conch diameter (mm)	Sex	Voxel size (mm)
*Allonautilus perforatus*	Indonesia	AMNH 125230	187	Unknown	0.084
		AMNH 125232	175	Unknown	0.083
*Allonautilus scrobiculatus*	Papua New Guinea	AMNH 81795	165	Male	0.083
		AMNH 131942	157	Unknown	0.079
*Nautilus belauensis*	Palau	AMNH 81905	204	Male	0.093
		AMNH 106854	214	Male	0.10
*Nautilus macromphalus*	New Caledonia	AMNH 131882	154	Unknown	0.071
		AMNH 131883	153	Unknown	0.0695
*Nautilus pompilius*	Papua New Guinea	AMNH 94937	152	Unknown	0.068
		AMNH 94931	147	Male	0.080
*Nautilus pompilius*	Philippines	NMNS PM 16245	177	Unknown	0.090
		NMNS PM 16273	164	Unknown	0.090
*Nautilus pompilius suluensis*	Philippines	AMNH 93768	121	Unknown	0.059
		AMNH 131880	128	Unknown	0.061
*Nautilus repertus*	Western Australia	AMNH 81270	216	Female	0.10
		AMNH 81273	212	Female	0.098
*Nautilus stenomphalus*	Lizard Island, Australia	AMNH 82062	148	Female	0.059
		AMNH 82072	159	Male	0.067

AMNH = American Museum of Natural History Fossil Invertebrates. NMNS PM = National Museum of Nature and Science, Japan.

**Figure 1:**
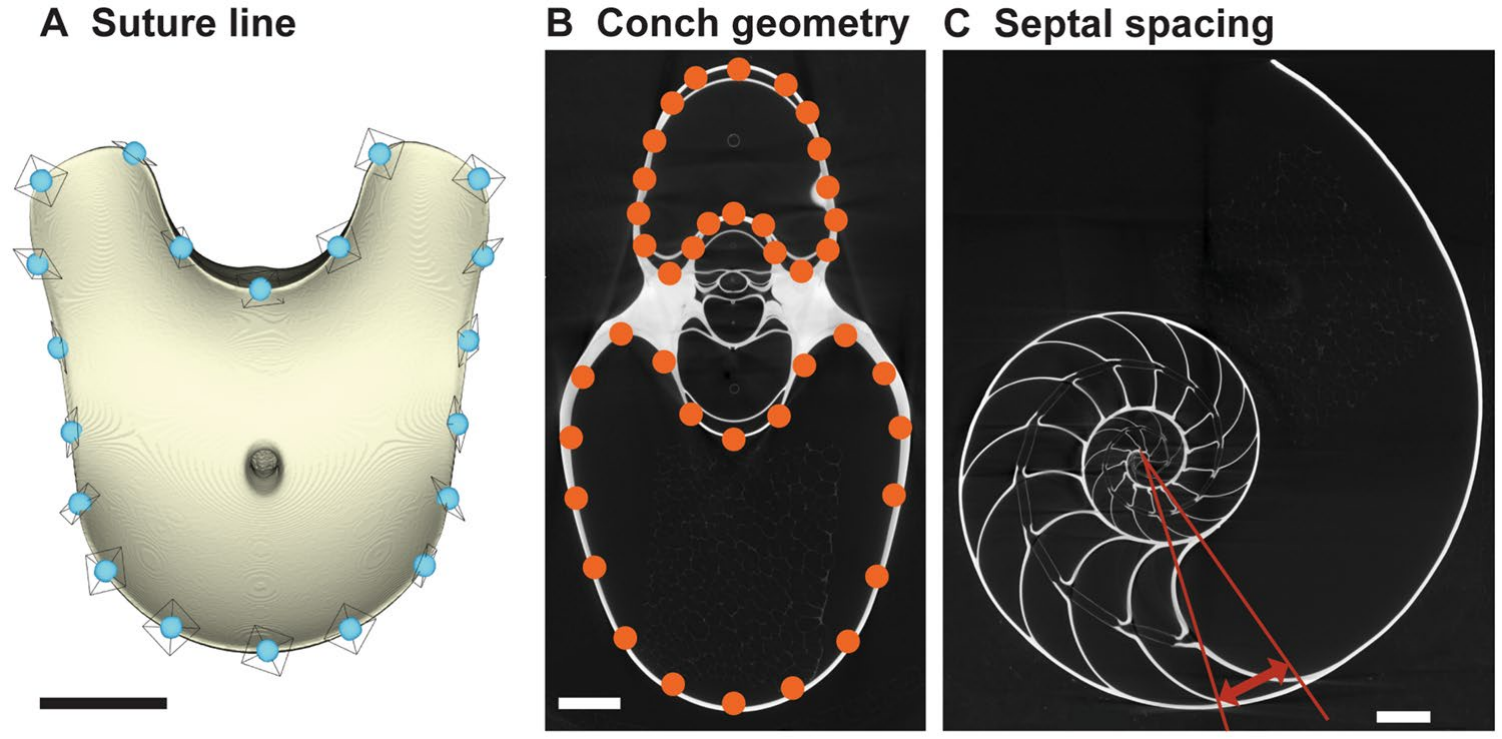
Analyzed morphological parameters (*Nautilus pompilius suluensis* Habe and Okutani, 1988; AMNH 93768). (**A**) Suture line (adoral view). A total of 20 landmarks were placed on the edge of the chamber. (**B**) Conch geometry (cross-sectional morphology). (**C**) Septal spacing (septal rotational angle). Scale bars = 10 mm.

We applied computed tomography in order to three-dimensionally reconstruct the conchs. A total of 16 specimens were CT-scanned at the Microscopy and Imaging Facility of the American Museum of Natural History and two specimens of *N. pompilius* from the Philippines were CT-scanned at the Port and Airport Research Institute, Japan. The image stacks obtained were segmented in Amira 2020 (Thermo Fisher Scientific) to export the interior surfaces of all chambers. Note that we excluded the earliest formed chamber (chamber 1) from our analyses due to potential artifacts resulting from limited scan resolution. To quantify the morphology of the suture line, we applied three-dimensional geometric morphometrics. Accordingly, we designated a total of 20 points of reference (so-called ‘landmarks’) with equidistant intervals along the edge of each chamber (i.e., suture line) in MATLAB (MathWorks). The landmarks for each chamber were slid along the outline of a reference individual to minimize the bending energy (sliding semi-landmark method^37^; Fig. 1A). Subsequently, we normalized and registered the landmark coordinates of each chamber according to its centroid size using the generalized Procrustes method^38^. The Procrustes residuals were then analyzed using principal components analysis (PCA) to reduce the dimensions of the data. We also examined two other morphological parameters—conch geometry and septal spacing. Conch geometry is the cross-sectional shape of a conch (Fig. 1B). We regard this parameter as the most commonly used character in the taxonomy of (mainly fossil) ectocochleate cephalopods. It is generally quantified using a linear morphometric approach^39–41^. We applied two-dimensional geometric morphometrics to analyze the conch geometry. First, we produced cross-sections of the conch every 45°, starting with the aperture (see Tajika and Klug^33^ for details), which enabled us to examine the morphology of ~ 20 ontogenetic points per specimen. Then, we designated a total of 40 landmarks with equidistant intervals along the shell on the whorl cross-section (Fig. 1B; compare Tajika et al.^41^). These landmarks were analyzed with the same methodology as the suture line. It should be noted that the conch geometry quantified here represents the morphology of the internal mold, the most common type of preservation in fossil ectocochleate cephalopods. Lastly, septal spacing was measured as the rotational angle between septa through ontogeny (Fig. 1C).

To visualize the similarity of each morphological character between species/populations, we constructed trees for the three morphological parameters using the neighbor-joining method^42^ and the Euclidian distances in PC space (based on all PCs). Comparisons of trees were carried out at three different ontogenetic stages. In this study, we used the conch diameters of 20 and 50 mm and maximal conch diameter as a proxy for the three different ontogenetic stages pre-hatching, middle ontogeny, and maturity. Accordingly, we picked two data points that are the closest to each of the conch diameters in each specimen. The specific ranges of each ontogenetic stage are approximately 18–21 mm (pre-hatching) and 45–55 mm (middle ontogeny; see Table 1 for maximal conch diameter of each specimen). Additionally, an analysis of variance (ANOVA) was performed to examine whether or not septal spacing significantly differs between species and populations. The analysis of variance was followed by multiple-comparison tests to determine which pairs of species/population are significantly different. These statistical tests were carried out at three different ontogenetic stages: (1) pre- hatching stage (conch diameter < 30 mm), (2) juvenile–submature stage (conch diameter ≥ 30 until the third chamber from the last), (3) mature stage (last two chambers). We conducted these analyses using the Statistics and Machine Learning Toolbox™ of MATLAB.

## Results

**Figure 2:**
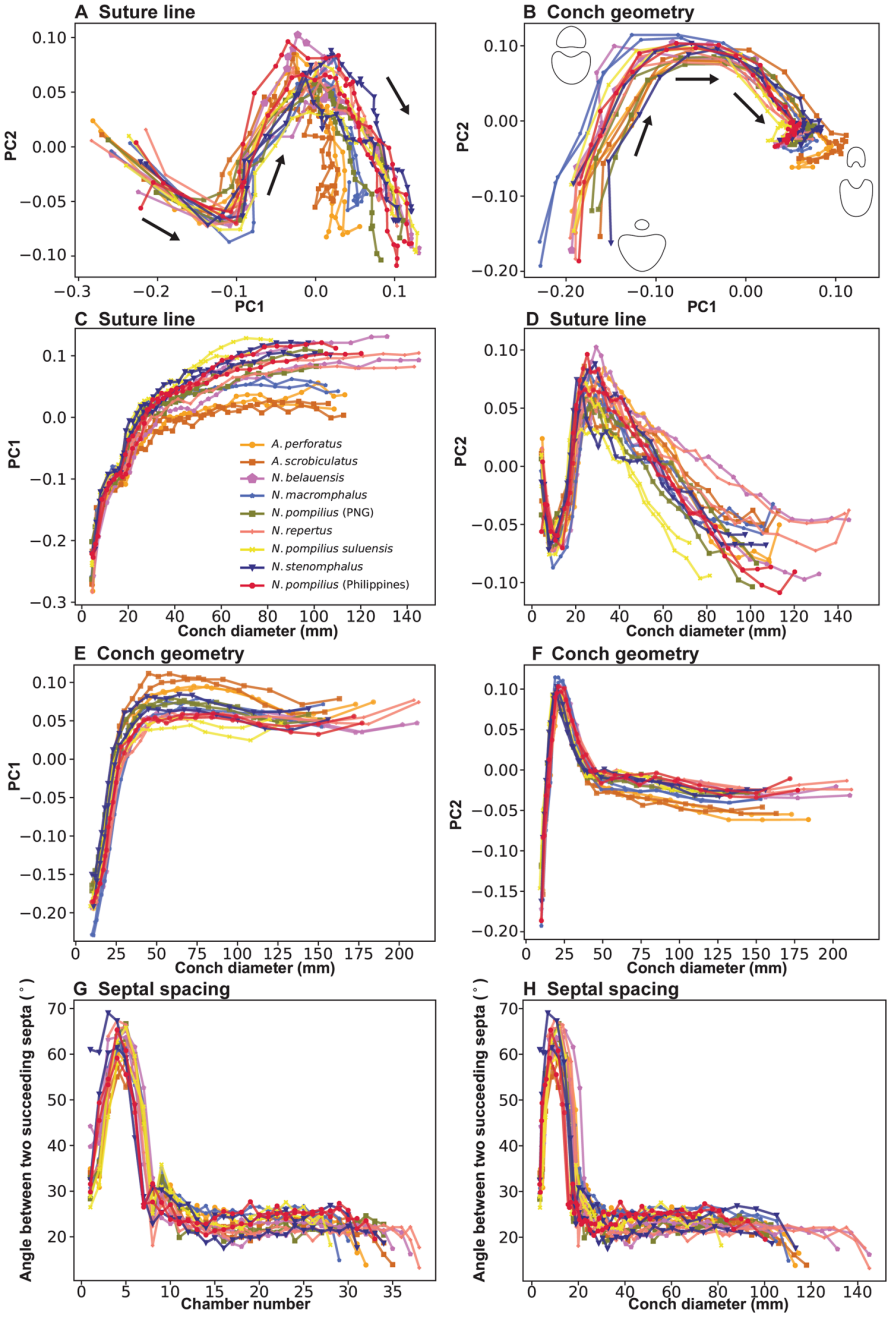
Results of morphological analysis. Points from the same individual are connected with a line. Arrows indicate the direction of growth. (**A**) PCA plot graph for sutural morphology. (**B**) PCA plot graph for conch geometry (cross-sectional morphology). (**C**) PC1 plotted against conch diameter for sutural morphology. (**D**) PC2 plotted against conch diameter for sutural morphology. (**E**) PC1 plotted against conch diameter for conch geometry. (**F**) PC2 plotted against conch diameter for conch geometry. (**G**) Septal spacing (septal rotational angle) plotted against chamber number. (**H**) Septal spacing (septal rotational angle) plotted against conch diameter (mm).

Figure 2A shows the results of PCA for suture line morphology. PC1 accounts for 63.0% of the total variance, PC2 for 20.6%, and PC3 for 3.9%. In the graph, all species share a similar ontogenetic pattern in the change of PC scores. PC1 increases rapidly until about a conch diameter of 30–40 mm after which it increases more gradually (Fig. 2C). PC2 shows a rapid decrease until about a conch diameter of 10 mm, followed by a rapid increase until a conch diameter of 30 mm (Fig. 2D). Then, it decreases steadily until the end of ontogeny. The ontogenetic changes of PC1 and PC2 indicate high morphological variation through ontogeny.

The results of PCA for conch geometry (cross-sectional morphology) are shown in Fig. 2B. In conch geometry, PC1 accounts for 65.1% of the total variance, PC2 for 24.0%, and PC3 for 6.4%. Similar to the morphology of the suture line, all species share a similar pattern in the change of PC scores for conch geometry through ontogeny. However, the patterns between suture line morphology and conch geometry differ. In conch geometry, PC1 increases until it reaches a plateau at a diameter of approximately 40 mm (Fig. 2E). PC2 rapidly increases until a diameter of 25 mm, followed by a sharp decrease; the decreasing trend becomes attenuated at a diameter of about 40 mm (Fig. 2F). The measurements of septal spacing are plotted in Fig. 2G,H. Septal spacing shows a steep increase followed by a steep decrease at chamber 7 or 8, which corresponds to the point of hatching^43^. Subsequently, septal spacing becomes relatively stable, with septa regularly spaced at 20–30°. Septal spacing decreases at the last septum or over the last few septa.

**Figure 3:**
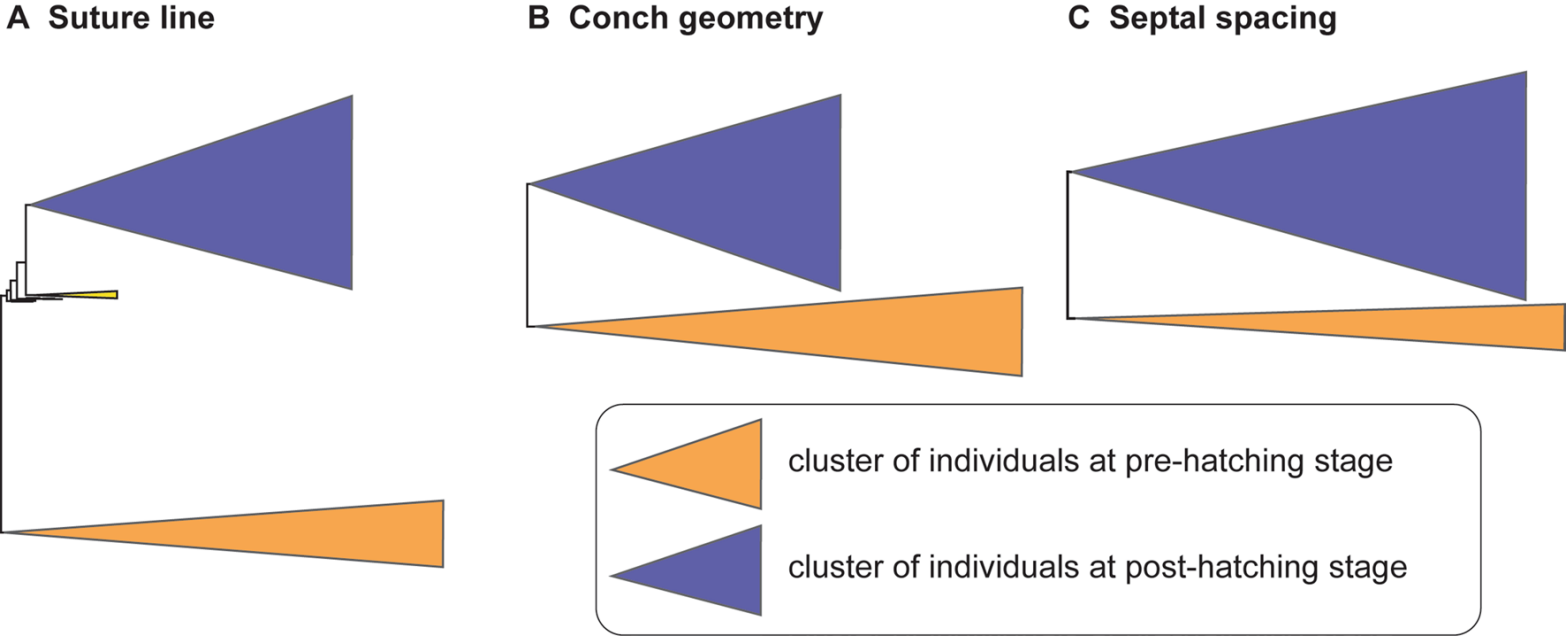
Simplified tree topologies for (**A**) the morphology of suture line, (**B**) conch geometry, and (**C**) septal spacing. The areas colored in orange and blue indicate the clusters at pre-hatching and post-hatching, respectively.

**Figure 4:**
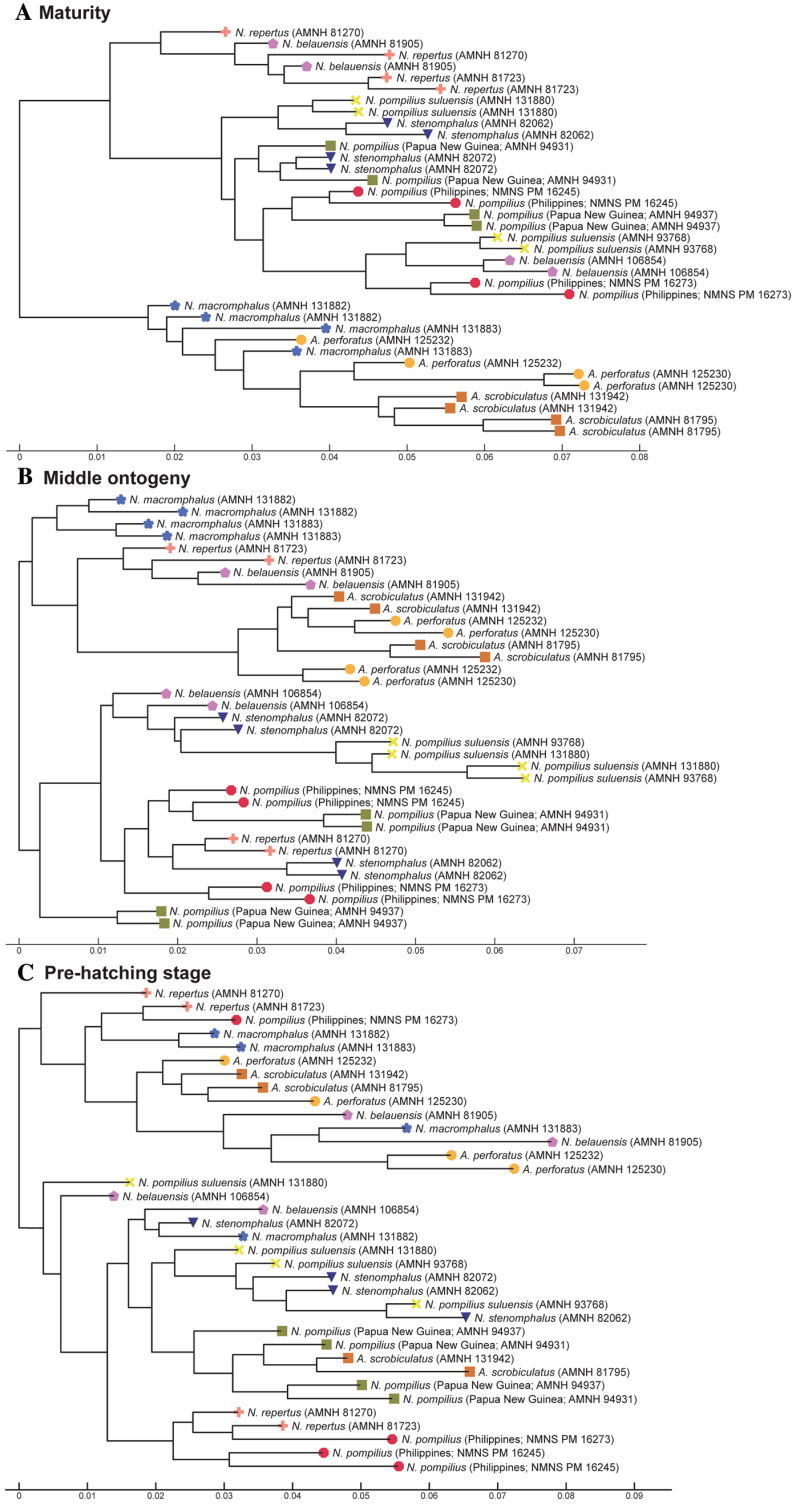
Trees for suture line. (**A**) Maturity (last two chambers). (**B**) Middle ontogeny (a diameter of about 50 mm). (**C**) Pre-hatching stage (a diameter of about 20 mm).

**Figure 5:**
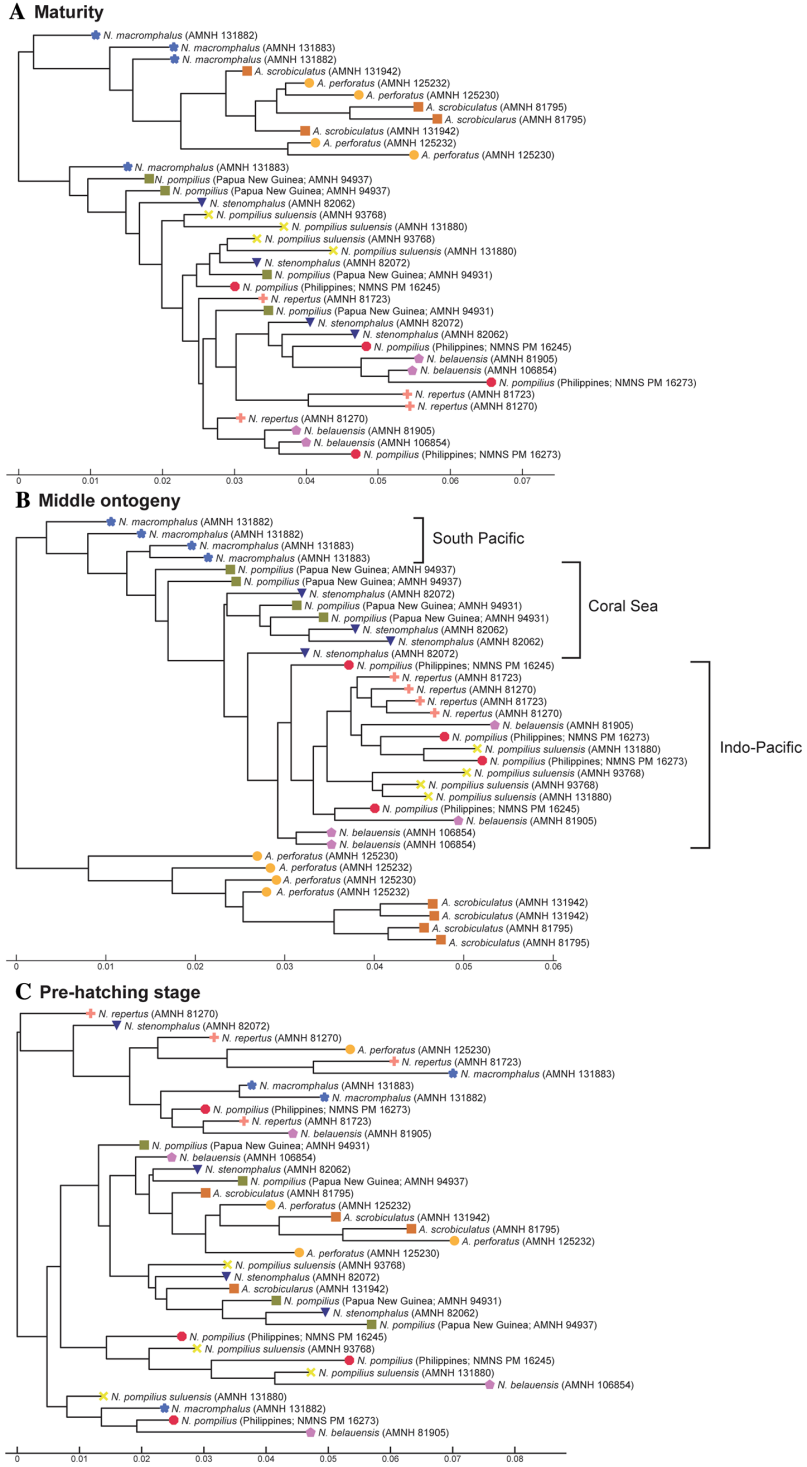
Trees for conch geometry (cross-sectional morphology). (**A**) Maturity (last two chambers). (**B**) Middle ontogeny (a diameter of about 50 mm). (**C**) Pre-hatching stage (a diameter of about 20 mm).

**Figure 6:**
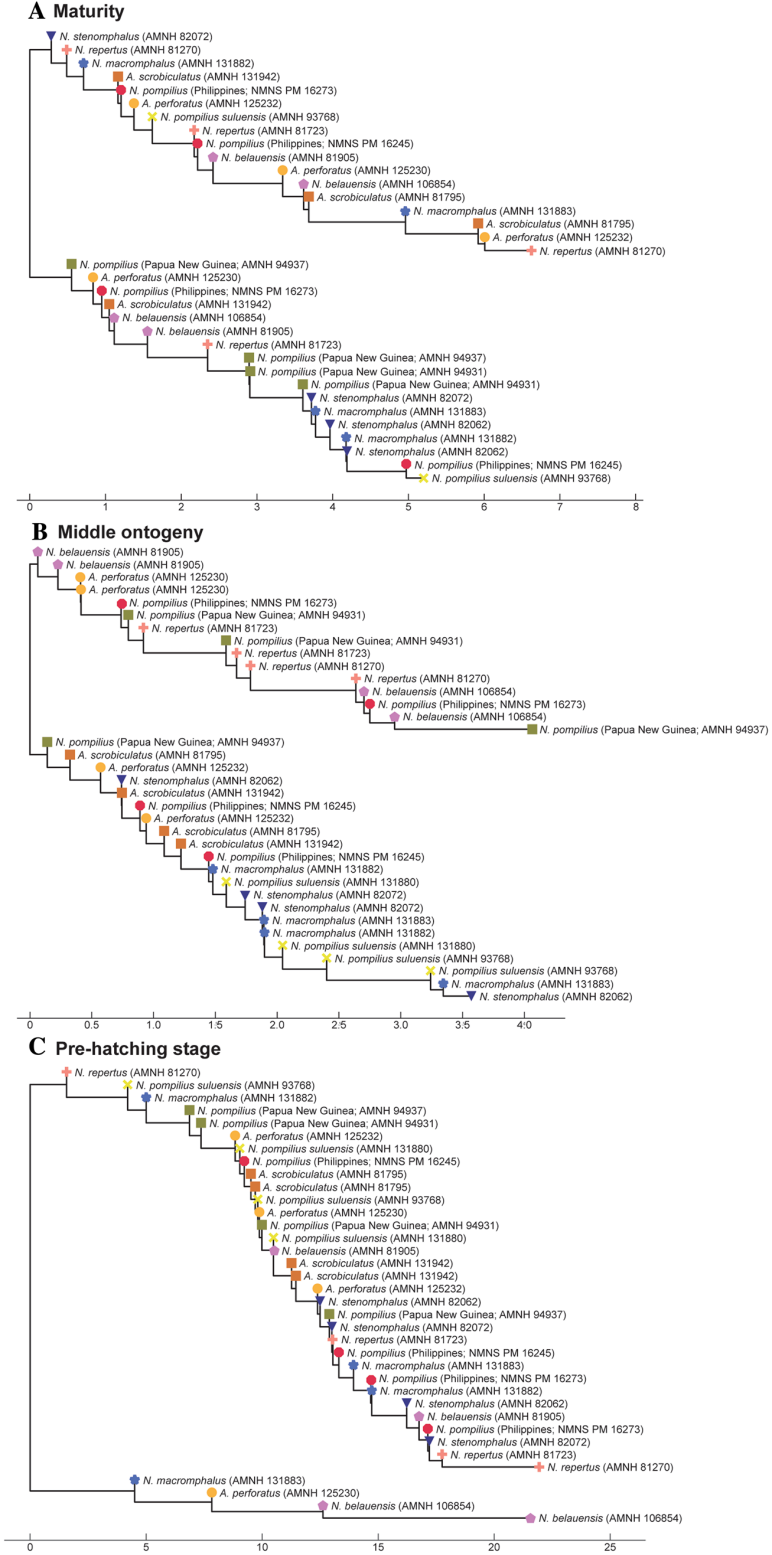
Trees for septal spacing. (**A**) Maturity (last two chambers. (**B**) Middle ontogeny (a diameter of about 50 mm). (**C**) Pre-hatching stage (a diameter of about 20 mm).

To illustrate the similarity among species, we constructed trees for each morphological character based on the PC scores (suture line, conch geometry) and measurements (septal spacing). As shown in Fig. 3, individuals at approximately the same ontogenetic stage form the largest clusters (pre-hatching versus post-hatching stages) rather than individuals of the same species. This suggests that morphological comparisons need to be made at a similar ontogenetic stage. Accordingly, we constructed trees based on the conch parameters using two chambers and two cross-sections at three different ontogenetic stages: diameters of about 20 mm (before hatching; Fig. 4), 50 mm (middle ontogeny; Fig. 5), and maturity (Fig. 6). In all examined characters, it is clear that the different species are less easily distinguished at the pre-hatching stage than at other ontogenetic stages (Figs. 4C, 5C, 6C). In addition, some taxa such as the two species of *Allonautilus* (*A. scrobiculatus* and *A. perforatus*) and *N. macromphalus* tend to form a single cluster, respectively, with a few exceptions for suture line and conch geometry (Figs. 4A,B and 5A,B). The morphological differences between species are visualized in Supplementary Video S1 and Supplementary Fig. S1. By contrast, taxa appear to be more randomly distributed with respect to septal spacing (Fig. 6). Figure 7 shows the range and distribution of septal spacing at different ontogenetic stages. The results of ANOVA reveal that there is no significant difference (p > 0.05; p-values available in Supplementary Table S1) at the pre-hatching (conch diameter < 30 mm) and adult stages but that the difference is significant at the middle ontogenetic stage (Supplementary Table S1). Multiple comparison tests demonstrate that a significant difference occurs both within- and between taxa at this stage (Fig. 7; Supplementary Table S1). In the following section, we will discuss the taxonomic implications in detail.

**﻿Figure 7:**
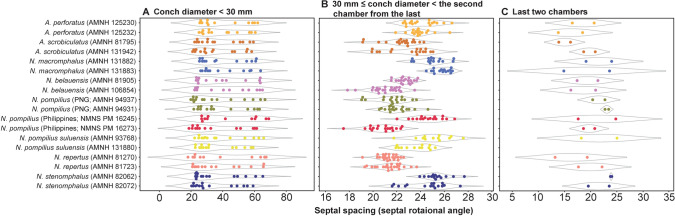
Range and distribution of septal spacing at different ontogenetic stages. (**A**) Early ontogeny (conch diameter < 30 mm). (**B**) Middle ontogeny (30 ≤ conch diameter < the second chamber from the last). (**C**) Late ontogeny (last two chambers).

## Discussion

### Potential artifacts

Although the application of computed tomography has become relatively common, there are some potential artifacts that may affect the results of our study. For instance, although the make-up of our specimens is entirely calcium carbonate with some organic remains, and the specimens were scanned at high resolution, the partial volume effect (PVE) results in a certain degree of error when reconstructing the volume^44^. In our study, the morphological analysis of the suture line is subject to this artifact because we needed to three-dimensionally reconstruct the chambers. As such, we estimated the difference between the actual and reconstructed conch volume using a silica sphere that was scanned together with the specimens. We found that the difference is ~ 10% in the examined specimens, implying that the reconstructed volume is not exactly precise. However, this is true for all specimens and, as a result, we consider that this artifact does not significantly affect our comparisons among the different specimens. Another possible error arises when there are some deposits such as cameral remains and broken shells within the conch. In this case, instead of relying on an automatic segmentation tool in Amira, we needed to manually segment the CT-images, which usually introduces some artifacts. In our specimens, we found a few chambers with infills. Nevertheless, we think that this artifact is quite negligible because we obtained a relatively distinctive ontogenetic pattern in our results without outliers (Fig. 2).

### Taxonomy

It should be noted that the validity of some of the studied nautilid taxa has been occasionally debated, although we explicitly excluded dubious species (sensu Saunders^45^) from our study. Most modern nautilid species were established based on their conch morphology, color pattern, and soft tissue anatomy. Yet, recent genetic studies have presented divergent views. For instance, Vandepas et al.^30^ studied *Nautilus belauenesis*, *N. repertus*, and *N. stenomphalus* using mitochondrial markers, which suggested that these species are simply variants of *N. pompilius*. Combosch et al.^27^ discovered that there is no genomic admixture between the geographic populations of *Nautilus* species in the South Pacific, Coral Sea, and Indo-Pacific. They concluded that previously described species are not concordant with their results and that there may be cryptic species that are geographically isolated (i.e., there are species that are only separable based on molecular data). In the following, we discuss whether such species with similar genetic distances show clear morphological differences.

### Similarity of the three morphological parameters among taxa

As briefly discussed in the results section, the three conch parameters differ in how useful they are in distinguishing species. For all three characters examined, the pre-hatching stage appears less useful than the other ontogenetic stages in distinguishing species and genera: all taxa tend to be more or less randomly distributed in the tree (Figs. 4C, 5C, 6C). This is especially conspicuous in septal spacing at this stage in which there is no significant difference in the mean value among taxa (Fig. 7A; Supplementary Table S1). With respect to the suture line (Fig. 4C), individuals within the same species at this stage tend to cluster with one another, with a few outliers. This implies that the intraspecific variation of suture line morphology is relatively high and that the morphology of one species may overlap with that of another before hatching. This also holds true for conch geometry at this stage (Fig. 5C).

In middle ontogeny (i.e., conch diameter ≈ 50 mm), the species are more clearly separated (Figs. 4B and 5B) except in septal spacing (Fig. 6B). When comparing the trees for suture line and conch geometry, the latter exhibits a clearer separation of species. For instance, although *Nautilus repertus*, *N. belauensis*, and *N. pompilius* appear in the two largest clusters in Fig. 4B, they are more or less separated in Fig. 5B. Also, both species of *Allonautilus* can more readily be distinguished using conch geometry than suture line at this ontogenetic stage. Our results are similar to the phylogeographic trees constructed by Combosch et al.^27^ and Vandepas et al.^30^ in that *Allonautilus scrobiculatus* and *N. macromphalus* form distinctive clusters. Furthermore, three geographic clusters of *Nautilus* from the South Pacific, Coral Sea, and Indo-Pacific shown by Combosch et al. are apparent in our results for conch geometry (Fig. 5B), although this separation is not visible for the suture line. This similarity with Combosch et al.^27^ implies that the five distinct clades that are potentially new *Nautilus* species may not, after all, be cryptic. As far as septal spacing is concerned, some species can be distinguished (Figs. 6B, 7B), but the fact that the ranges of some species—even genera—overlap, with high intraspecific variation (e.g., *N. belauensis* in Fig. 7B) suggests that septal spacing is not useful in middle ontogeny.

As in middle ontogeny, the suture line and conch geometry appear to be better characters than septal spacing to distinguish species in late ontogeny (Figs. 4A, 5A, 6A). Regarding the suture line, species are better separated at maturity than at the juvenile stage. *Allonautilus perforatus* and *A. scrobiculatus* are distinguished from one another although one specimen of *A. perforatus* is morphologically closer to *Nautilus macromphalus* than to *A. scrobiculatus* (Fig. 4A). The mixture of *A. perforatus* and *N. macromphalus* suggests that the morphological difference in suture line between the two taxa is not as conspicuous as that of external conch morphology. All *Nautilus* species except *N. macromphalus* are difficult to separate out. Regarding conch geometry, species do not separate out as readily as at the middle ontogenetic stage, although there are some species-specific patterns (Fig. 5A). The species of *Allonautilus* form a cluster and one individual of *N. macromphalus* lies outside the cluster for *N. macromphalus* and both species of *Allonautilus* (Fig. 5A). As far as the other *Nautilus* species are concerned, the morphological variation of each species seems to hamper separation in late ontogeny. Septal spacing at maturity does not significantly differ in any species (Figs. 6A, 7C), suggesting that septal spacing is not a useful diagnostic character throughout ontogeny. This indicates that septal formation may not be affected by environmental factors such as food supply and water chemistry, resulting from the different habitats of modern nautilids.

### Morphological similarity and phylogeny

Some researchers have suggested that the suture line may be a better character than conch geometry for reconstructing the phylogeny of ectocochleate cephalopods as the latter is highly homoplastic (see Klug and Hoffmann^46^ and references therein). This is usually the case for at/above generic level in ammonoids. Although it is not our purpose to reconstruct the phylogeny of modern nautilids using morphological characters, we briefly discuss our result in the context of phylogeny.

As mentioned above, all modern nautilid species were established based on morphology, and thus morphological differences should be visible among species. However, most species cannot be distinguished in our results at early and adult ontogenetic stages, with the exception of *Nautilus macromphalus* and the two species of *Allonautilus*. The differences in *Nautilus* species are apparent only for conch geometry in middle ontogeny. Although the reason behind this is unclear, it may be rooted in morphological constraints at maturity regarding reproductions, which are shared by different species (see the discussion of morphogenetic countdown at maturity in Tajika and Klug^33^ and Seilacher and Gunji^47^). Additionally, species specific characters may not develop before hatching. When comparing our results and previous molecular studies, the tree based on conch geometry in middle ontogeny is most similar to the phylogeographic tree of Vandepas et al.^30^ and Combosch et al.^27^ (for a simiplified phylogenetic tree by Combosch et al.^27^ see Supplementary Fig. S2). These results suggest that conch morphology—particularly in middle ontogeny—may yield a robust phylogeny similar to that reconstructed using molecular data. Further research with more specimens and more data (e.g., sex, habitat) and more rigorous methodology for phylogenetic reconstruction are needed.

### Implications for taxonomy of fossil ectocochleate cephalopods

Our results revealed that (1) intraspecific variation is higher than interspecific variation at a particular ontogenetic stage, (2) modern nautilid species can be separated out based on conch geometry (cross-sectional morphology) in middle ontogeny, and (3) it is difficult to distinguish modern species based on the suture line with the exception of *Nautilus* versus *Allonautilus*.

We suspect that our results also apply to fossil nautilids. Tajika et al.^41^ investigated the ontogeny of Late Cretaceous *Eutrephoceras dekayi* (Morton, 1834) and modern *Nautilus pompilius*. They discovered a similar ontogenetic pattern in whorl expansion rate, whorl width index, and septal spacing index between the two taxa despite the fact that the two taxa lived in different environments at different times (i.e., *E. dekayi* in shallow water epicontinental seas in North America during the Late Cretaceous vs. *N. pompilius* in much deeper water on steep forereef slopes in the Philippines today). Their similar ontogenetic trajectories in morphological space may suggest a similar evolutionary heritage rather than an adaptation to a particular environment. Thus, we presume that our results may be applicable to at least some groups of fossil nautilids. As mentioned above, however, detailed morphological studies are largely lacking in fossil nautilids with few exceptions^41,48,49^ and, therefore, additional data on the conch morphology (e.g., siphuncular position, siphunclular thickness, soft tissue attachment, classical conch parameters) through ontogeny are needed to further discuss the potential application in fossil forms.

In contrast to nautilids, ammonoids are known to possess a remarkably diverse array of conch shapes. They also exhibit high intraspecific variation, possibly in response to variation in their environment^50–53^. As a result, the ontogenetic trajectory in morphological space of ammonoids is highly variable^54–57^. Taking these observations into consideration, it implies that the various patterns of conch morphology in ammonoids also differ from those in modern nautilids. Although the phenotypic plasticity and ecophenotypic variability of the suture line in ammonoids have never been studied in detail to our knowledge, we cannot determine whether the suture line is the most useful character in distinguishing ammonoid species with our current data. As evidenced by our results, even a slight difference in conch diameter can significantly affect the suture line. We, therefore, strongly suggest examining the suture line at the same conch diameter. Our data also demonstrate that even distantly related nautilid species (e.g., *Nautilus macromphalus* and *Allonautilus scrobiculatus*) exhibit similar suture lines and, thus, we suggest caution in using the suture line as the only character in taxonomic studies.

## Supplementary Information

Supplementary Information 1.

Supplementary Note.

Supplementary Video S1.

Supplementary Figure S1.

Supplementary Figure S2.

Supplementary Table S1.

## Data Availability

Results of statistical tests and raw data are available in Supplementary Table S1.
