# Supplementary material for: Significance of the suture line in cephalopod taxonomy revealed by 3D morphometrics in the modern nautilids Nautilus and Allonautilus
Source: Sci Rep. 2021 Aug 24;11:17114. doi: 10.1038/s41598-021-96611-1 (PMC8384854; doi:10.1038/s41598-021-96611-1)
Supplement: Supplementary file 1 — Supplementary Information 1. [file 41598_2021_96611_MOESM1_ESM.docx]

Supplementary Note. Brief descriptions of studied modern nautilid species.

Supplementary Video S1. Visualization of morphological difference in the last suture line between *Allonautilus scrobiculatus* (Papua New Guinea) and *Nautilus pompilius* (Philippines).

Supplementary Fig. S1. Visualization of morphological difference in conch geometry at middle ontogeny in all examined species.

Supplementary Fig. S2. Simplified phylogenetic tree (modified after Combosch et al. 2017)

Supplementary Table S1. Results of statistical tests and raw data.
