# Supplementary material for: Significance of the suture line in cephalopod taxonomy revealed by 3D morphometrics in the modern nautilids Nautilus and Allonautilus
Source: Sci Rep. 2021 Aug 24;11:17114. doi: 10.1038/s41598-021-96611-1 (PMC8384854; doi:10.1038/s41598-021-96611-1)
Supplement: Supplementary file 2 — Supplementary Note. [file 41598_2021_96611_MOESM2_ESM.docx]

**Significance of the suture line in cephalopod taxonomy revealed by 3D morphometrics in the modern nautilids *Nautilus* and *Allonautilus***

Amane Tajika, Naoki Morimoto, Neil H. Landman

Supplementary Note

***Allonautilus perforatus* (Conrad, 1847)**

The conch diameter measures ~180 mm at maturity with a wide umbilicus and sharp umbilical shoulder. The whorl section is somewhat trapezoidal with a broadly rounded venter. According to Ward and Saunders ^1^, the shell bears reticulate ornament prior to the nepionic constriction. There are moderate undulating ribs on the flanks of the adult body chamber. The soft tissue of this species has not been found. This species may be geographically separated from *A. scrobiculatus* ^1^.

***Allonautilus scrobiculatus* (Sowerby, 1849)**

The conch diameter measures ~180 mm at maturity ^2^ with a wide umbilicus and sharp umbilical shoulder. The whorl section is somewhat trapezoidal with a broadly rounded venter, flat flank, and vertical umbilical wall. *Allonautilus scrobiculatus* has conch parameters similar to *A. perforatus^1^* but differs in having a smooth shell on the flanks of the adult body chamber.

***Nautilus belauensis* Saunders, 1981**

The conch diameter measures ~200 mm at maturity ^2^. The umbilicus is covered by a callus and is not visible in most of ontogeny. The conch morphology is similar to *N. pompilius*. This species is characterized by its large conch diameter at maturity.

***Nautilus macromphalus* Sowerby, 1849**

The conch diameter measures ~162 mm at maturity ^2^. This species has a relatively wide umbilicus with only some callus. The whorl section is slightly more broadly rounded than *N. pompilius*. *N. macomphalus* differs from *A. scrobiculatus and A. perforatus* in having a smaller umbilicus and more rounded umbilical shoulder.

***Nautilus pompilius* Linnaeus, 1758**

The average conch diameter varies, depending on the geographic population (~125–200 mm at maturity) ^2^. A small umbilicus is covered by a callus and is not visible in most of ontogeny. The whorl section appears to be slightly less wide than other modern nautilid species ^1^ and broadly rounded. The umbilical wall is more narrowly rounded than in *N. macromphalus*.

***Nautilus pompilius suluensis* Habe and Okutani, 1988**

This species is characterized by a small conch diameter at maturity (~100 mm). The conch morphology is superficially similar to *N. pompilius.* The umbilicus is covered by a callus and is not visible. The coloration is more purplish than reddish ^3^, which differs from *N. pompilius*. This species is known only from the Sulu Sea in the Philippines.

***Nautilus repertus* Iredale, 1944**

This species is characterized by a large conch diameter at maturity (~220 mm). The conch morphology is superficially similar to that of *N. pompilius* but *N. repertus* bears undulating ribs on the flanks of the adult body chamber. The umbilicus is covered by a callus and is not visible. There is white coloration in the umbilical area. This species is known only from Western Australia.

***Nautilus stenomphalus* Sowerby, 1848**

The conch diameter measures ~165 mm at maturity. The species has white coloration in the umbilical area ^4^. Some individuals lack an umbilical callus. The conch morphology is somewhat similar to *N. pompilius*. *N. stenomphalus* can be distinguished on the basis of hood texture. This species is known only from Lizard Island in Australia.

1 Ward, P. D. & Saunders, W. B. *Allonautilus*: a new genus of living nautiloid cephalopod and its bearing on phylogeny of the Nautilida. *Journal of Paleontology* **71**, 1054-1064 (1997).

2 Saunders, W. B. in *Nautilus The Biology and Paleobiology of a Living Fossil* (eds W Bruce Saunders & N H Landman) 35-52 (Springer, 1987).

3 Habe, T. & Okutani, T. A new subspecies of living *Nautilus* (Cephalopoda: Nautiloidea) from the Sulu Sea. *Venus (Japanese Journal of Malacology)* **47**, 91-94 (1988).

4 Saunders, W. & Ward, P. Sympatric occurrence of living *Nautilus* (*N. pompilius* and *N. stenomphalus*) on the Great Barrier Reef, Australia. *Nautilus* **101**, 188-193 (1987).
