# Supplementary figures and images for: Significance of the suture line in cephalopod taxonomy revealed by 3D morphometrics in the modern nautilids Nautilus and Allonautilus
Source: Sci Rep. 2021 Aug 24;11:17114. doi: 10.1038/s41598-021-96611-1 (PMC8384854; doi:10.1038/s41598-021-96611-1)

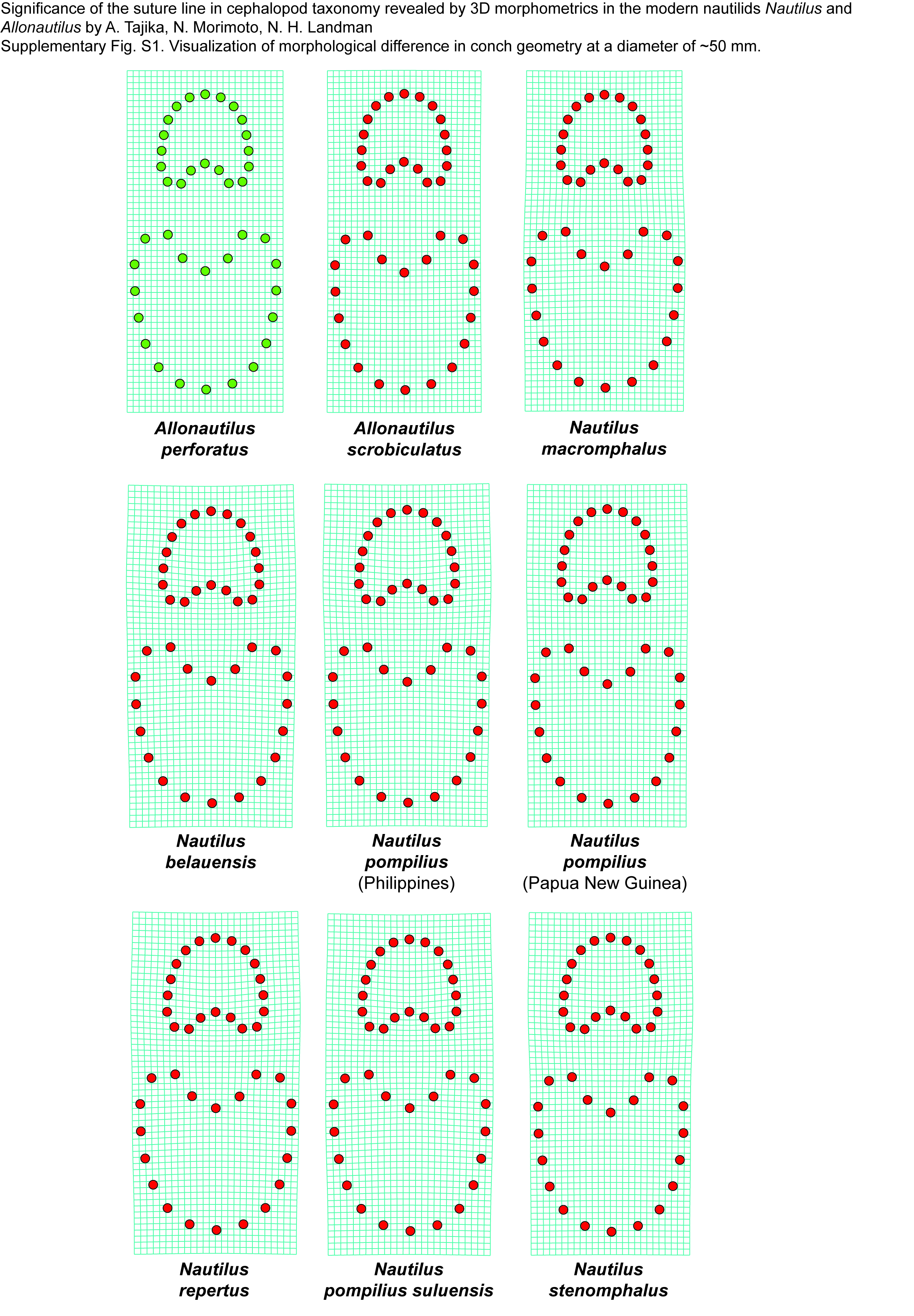

Supplement: Supplementary file 4 — Supplementary Figure S1. [file 41598_2021_96611_MOESM4_ESM.tif]

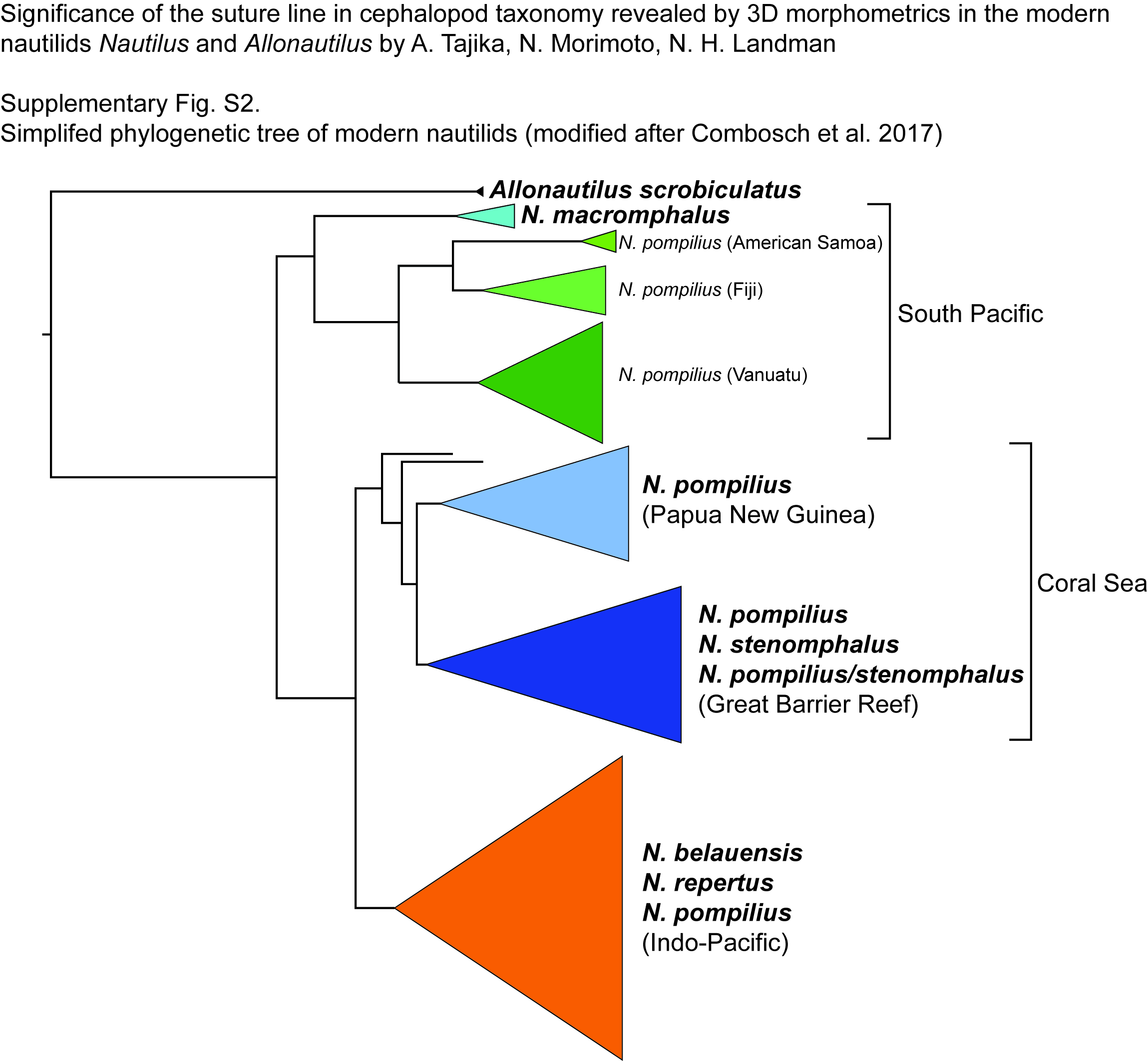

Supplement: Supplementary file 5 — Supplementary Figure S2. [file 41598_2021_96611_MOESM5_ESM.tif]
